# Pharmaceutical industry payments to authors of neurology clinical practice guidelines in Japan: A cross‐sectional study

**DOI:** 10.1002/hsr2.70101

**Published:** 2024-09-25

**Authors:** Anju Murayama, Yuki Senoo

**Affiliations:** ^1^ School of Medicine Tohoku University Sendai Japan; ^2^ Department of Population Health Science and Policy Icahn School of Medicine at Mount Sinai New York New York USA; ^3^ Higashi Totsuka Memorial Hospital Yokohama Japan

**Keywords:** clinical guidelines, conflicts of interest, health policy, medical ethics, transparency

## 1 INTRODUCTION

Over the past three decades, clinical practice guidelines (CPGs) have increasingly played instrumental roles in standardizing the diagnostic and treatment processes based on the best available evidence. CPGs are now essential tools for endorsing evidence‐based medicine in clinical practice.^1^, ^2^ However, the trustworthiness of CPGs could be compromised by financial conflicts of interest (COIs) between CPG authors and the pharmaceutical industry. Over the past decade, accumulating evidence has shown prevalent financial relationships between CPG authors and the healthcare industry.^3^, ^4^, ^5^, ^6^, ^7^, ^8^, ^9^ While not all financial interactions necessarily lead to problematic relationships or harmful influences on patients and physicians’ clinical practice, some can introduce bias into CPG recommendations, potentially compromising patient‐centered care.^2^, ^10^, ^11^ A recent systematic review indicated that CPGs and advisory committee reports with COIs were more likely to make favorable recommendations for pharmaceutical companies.^12^

To mitigate concerns about the undue influence of the healthcare industry on CPG recommendations, many national and international professional organizations have implemented strict COI management policies for trustworthy CPG development.^1^, ^2^, ^5^, ^11^, ^13^, ^14^, ^15^ Given the significant impact of CPGs on patients, clinicians, and other stakeholders, stringent COI management—including full disclosure, minimization of COIs among authors and organizations, and the appointment of COI‐free chairpersons for CPGs—is essential. This approach could create reliable CPGs and advances patient‐centered care in the field of neurology and beyond.^2^, ^3^, ^5^, ^11^, ^16^, ^17^, ^18^ Nevertheless, the extent of financial COIs among neurology CPG authors has not been thoroughly investigated to date. Utilizing a publicly accessible database containing personal payments to physicians from pharmaceutical companies, this study aimed to evaluate the fraction and size of financial COIs among neurology CPG authors in Japan.

## 2 METHODS

This retrospective study analyzed the size and proportion of personal payments made by pharmaceutical companies to all authors of CPGs published by the Japanese Society of Neurology between 2016 and 2020. All pharmaceutical companies affiliated with the Japan Pharmaceutical Manufacturers Association (JPMA), the largest pharmaceutical industry trade organization, are required to disclose payments made to physicians for activities such as lectures, consultancy services, and manuscript and pamphlet writing, including the names of individual physicians, on their respective company websites.^19^ However, due to the decentralized nature of these disclosures, the accessibility of payment information across companies has been substantially limited in Japan. To enhance public access to payment disclosures, a research organization independently collects payment records from all pharmaceutical companies affiliated with the JPMA and publishes them in a searchable online database (https://yenfordocs.jp/). The latest version of this database contained payment data from 2016 to 2020. We retrieved data on payments for lecturing, consulting, and writing compensations made to the CPG authors during this period from the payment database following the steps.

First, a comprehensive list of all CPG authors was compiled and subsequently exported as a Comma‐Separated Values file. Second, we searched for the names of CPG authors in the payment database and extracted personal payments to physicians whose names matched those of the CPG authors using Python programming, as previously described.^20^, ^21^, ^22^ Third, we manually reviewed all extracted payment records to identify and remove any payments made to physicians with similar names to the CPG authors but who were different individuals.^21^ Finally, 15 CPG authors were randomly selected (representing 5.3% of all authors), and their names were manually searched for in the payment database. The extracted data obtained with Python was then checked for accuracy and completeness.

The total amounts of payments and the number of CPG authors receiving payments were calculated. Descriptive analyses, including mean, standard deviation (SD), median, and interquartile range (IQR), were performed on the payment data collected from the companies between 2016 and 2020.

As this study was a retrospective analysis of publicly available data and met the criteria for nonhuman subjects research, institutional board approval was not required.

## 3 RESULTS

We identified 284 unique authors from the 10 CPGs published by the Japanese Society of Neurology between 2016 and 2020. Among these authors, 34 (12.0%) contributed to the development of two different CPGs, 241 (84.9%) were male physicians, and 73 (25.7%) held full professorships at their affiliated universities.

Of these authors, 236 (83.1%) received one or more personal payments from pharmaceutical companies between 2016 and 2020 (Table 1). The total amount of payments to 273 authors was $13.9 million, encompassing 14,596 transactions over the 5 years. The mean and median personal payments per author were $49,274 (SD: $81,146) and $15,255 (IQR: $1,138–$58,737), respectively. Over the 5 years, 28.2%, 16.2%, and 4.6% of authors received more than $50,000, $100,000, and $250,000, respectively. All 10 CPG chairpersons and nine vice chairpersons received personal payments, with a mean of $118,450 (standard deviation: $153,378). The mean payment amounts were significantly higher for CPG chairs and vice chairpersons than for other authors ($118,450 vs. $44,593, *p* < 0.001 in the Mann‐Whitney *U* test).

**Table 1:** Summary of personal payments to Japanese neurology guideline authors between 2016 and 2020.

Variables	Values
Total amount of payments, $	13,993,788
Mean payments per author (standard deviation), $	49,274 (81,146)
Median payments per author (interquartile range), $	15,255 (1138–58,737)
Maximum, $	616,579
Authors with payments (*N* = 284), n (%)	
Any payments	236 (83.1)
>$10,000	159 (56.0)
>$50,000	80 (28.2)
>$100,000	46 (16.2)
>$250,000	13 (4.6)
Top five companies making the largest payment amounts (%), $	
Eisai	1,857,121 (13.3)
Takeda Pharmaceutical	1,434,193 (10.2)
Daiichi Sankyo	1,416,524 (10.1)
Sumitomo Pharma	1,123,221 (8.0)
Novartis Pharma	1,060,114 (7.6)

Table 2 describes the payments by guideline. Of the 10 CPGs, all had more than 50% of their authors receiving personal payments, with percentages ranging from 72.6% to 100%. All authors of the spinocerebellar degeneration and multiple system atrophy CPG received payments from the companies. The mean personal payments were highest for the Parkinson's disease CPG ($160,441), followed by epilepsy ($78,110), dementia ($72,431), and spinocerebellar degeneration and multiple system atrophy ($44,989).

**Table 2:** Personal payments to authors of neurology clinical practice guidelines published by the Japanese Society of Neurology.

Diseases targeted by clinical practice guidelines (publication year)	Number of authors, n	Number of authors receiving payments (%), n	Total amounts of payments, $	Mean payment values (standard deviation), $	Median payment values (interquartile range), $	Maximum payment values per author, $
Myotonic dystrophy (2020)	32	25 (78.1)	474,953	14,842 (26,378)	2724 (313–16,592)	117,092
Prions diseases (2020)	27	22 (81.5)	774,907	28,700 (34,513)	10,320 (483–46,057)	127,784
HTLV‐1 associated myelopathy (2019)	51	37 (72.6)	1,650,249	32,358 (80,067)	6856 (0 – 33,413)	426,273
Dystonia (2018)	26	20 (76.9)	920,439	35,401 (63,483)	8203 (936–41,711)	269,901
Spinocerebellar degeneration and multiple system atrophy (2018)	23	23 (100)	1,034,751	44,989 (48,592)	19,859 (4,675–87,867)	148,035
Epilepsy (2018)	21	18 (85.7)	1,640,303	78,110 (84,511)	43,284 (13,014–114,702)	273,311
Parkinson's disease (2018)	19	18 (94.7)	3,048,371	160,441 (152,860)	111,640 (34,124–269,927)	616,579
Herpes simplex encephalitis (2017)	18	14 (77.8)	332,863	18,492 (22,461)	9913 (1,043–30,705)	81,116
Dementia (2017)	62	55 (88.7)	4,490,709	72,431 (90,761)	29,718 (6,867–111,886)	332,147
Multiple sclerosis and neuromyelitis optica (2017)	39	33 (84.6)	1,645,834	42,201 (51,833)	13,345 (1,579–88,059)	203,598

## 4 DISCUSSION

This study represents the first comprehensive analysis of personal payments to all neurology CPG authors from major pharmaceutical companies in Japan. We demonstrated that over 80% of neurology CPG authors received nearly $14.0 million in personal payments over the 5 years from 2016 to 2020. These payments were for lectures at company‐sponsored events, consulting services, and supervising pamphlets about the companies’ products distributed to physicians and patients. Notably, all CPG chairpersons and vice chairpersons had substantially financial ties with pharmaceutical companies.

These close financial relationships between Japanese neurology CPG authors and pharmaceutical companies raise concerns about the Japanese Society of Neurology's management of financial COIs for CPG authors. This situation may also pose a risk to the credibility and integrity of neurology CPGs in Japan. The high proportions of CPG authors receiving personal payments and the substantial payments to CPG chairpersons during the CPG development and/or a few years after CPG publication indicate clear deviations from international COI policies. According to recommendations by the U.S. National Academy of Medicine and the Guidelines International Network,^1^, ^2^ medical societies and organizations responsible for producing CPGs should maintain a majority of authors free from financial COIs and appoint chairpersons without such conflicts. However, our findings reveal that none of the CPGs developed by the Japanese Society of Neurology met these recommendations.

The deviations of Japanese CPGs from international COI policies and widespread COIs among CPG authors are not unique to neurology CPGs but are also evident across specialties in Japan,^3^, ^4^, ^5^, ^6^, ^7^, ^18^, ^23^, ^24^, ^25^, ^26^, ^27^, ^28^, ^29^ indicating that neurology is not an exception to a specialty that is free from potential industry influence and bias. Studies reported that the proportion of CPG authors with financial COIs ranged from 78.2% in oncology^30^ to 91.3%–100% in rheumatology.^4^, ^24^ These high proportions may be attributed to less transparent and rigorous COI management policies among Japanese professional medical societies,^4^, ^5^ including the Japanese Society of Neurology. The Japanese Society of Neurology only required authors to declare payments exceeding $4,682 (500,000 Japanese yen) per year per company for activities such as lecturing, consulting, and writing. Thus, payments below this threshold were not mandated to be declared, despite the majority of US and European medical societies requiring disclosure of all payments regardless of amount.^14^ Given the significant influence of CPGs on clinical practice and patient care, more transparent and rigorous COI management policies as well as enforcement of the policies are essential for future CPGs developed by the Japanese Society of Neurology.

This study has several limitations. First, the payment data were extracted from a secondary database maintained by an independent research organization. As the organization acknowledged, the study cannot rule out the possibility of errors or misreporting in the payment data reported in the database. However, the organization actively addresses potential inaccuracies by encouraging users to report errors, which are then verified and corrected when necessary. Additionally, another potential source of inaccuracy is the disclosure practices of the companies themselves. Since the payment disclosures are based on voluntary guidance issued by the JPMA, there is no penalty for deviating from the guidance or for inaccurate reporting in Japan. Furthermore, no independent system exists to verify the accuracy of the payment records disclosed by the companies. While this could lead to underreporting of payment records, it is unlikely to result in overreporting. Therefore, this study may underestimate but not overestimate the financial relationships between the CPG authors and pharmaceutical companies. Second, payments from pharmaceutical companies not affiliated with the JPMA were not disclosed, preventing assessment of the full extent of financial relationships between CPG authors and non‐JPMA affiliated companies. However, since JPMA member companies account for more than 80% of the market share for prescription drugs in Japan,^31^ the impact of financial relationships between the authors and non‐JPMA affiliated companies is likely minimized in this study. Finally, the JPMA only mandates their member companies to disclose payments for lecturing, consulting, and writing compensations at individual physician level, and other types of payments such as travel, accommodation, meal, small gifts, trial registration, and royalty/license fees. Therefore, the CPG authors would have received other payments that were not measured in this study.

## AUTHOR CONTRIBUTIONS

**Anju Murayama**: Conceptualization; Investigation; Funding acquisition; Writing—original draft; Writing—review and editing; Visualization; Methodology; Software; Formal analysis; Project administration; Resources; Supervision. **Yuki Senoo**: Conceptualization; Writing—original draft; Writing—review and editing.

## CONFLICT OF INTEREST STATEMENT

The authors declare no conflict of interest.

## ETHICS STATEMENT

As this study was a retrospective analysis of publicly available data and met the definition of nonhuman subjects research, no institutional board review and approval were required. This study followed the Strengthening the Reporting of Observational Studies in Epidemiology (STROBE) guideline. During the preparation of this work, the authors used ChatGPT version 4.0 to check and correct grammatical and spelling errors. After using this tool, the authors carefully reviewed and edited the content as needed and take full responsibility for the content of the publication.

## TRANSPARENCY STATEMENT

The lead author Anju Murayama affirms that this manuscript is an honest, accurate, and transparent account of the study being reported; that no important aspects of the study have been omitted; and that any discrepancies from the study as planned (and, if relevant, registered) have been explained.

## Data Availability

The data that support the findings of this study are available on request from the corresponding author. The data are not publicly available due to privacy or ethical restrictions. All data used in this study are available from Yen For Docs database (https://yenfordocs.jp/) and each pharmaceutical companies belonging to the Japan Pharmaceutical Manufacturers Association. Due to privacy protection of guideline authors, the datasets generated and/or analyzed during the current study are available from the corresponding author on reasonable request.
